# Supplementary material for: Identification of Freezing-Responsive microRNAs and Their Targets in Chinese Jujube by Small RNA and Degradome Sequencing
Source: Int J Mol Sci. 2026 Sep 4;27(17):7912. doi: 10.3390/ijms27177912 (PMC13566890; doi:10.3390/ijms27177912)
Supplement: Supplementary file 1 [file ijms-27-07912-s001.zip › Supplementary Material S5.pdf]

Table S5. The detailed information of DEG miRNAs between JCK and JT

ID	Pvalue	FDR	log2FC	regulated
bcy-miR156	2.64E-06	1.10E-05	3.413721503	up
bgy-miR156	2.64E-06	1.10E-05	3.413721503	up
cme-miR156a	0.003694689	0.010480852	5.516131617	up
cpa-miR156e	2.64E-06	1.10E-05	3.413721503	up
eun-miR162-3p	8.05E-09	5.89E-08	-1.332251957	down
fve-miR156g-3p	0.00031716	0.001102132	6.931423869	up
fve-miR156g-5p	3.41E-07	1.69E-06	3.251752874	up
fve-miR162-3p	8.05E-09	5.89E-08	-1.332251957	down
gma-miR162c	8.05E-09	5.89E-08	-1.332251957	down
gma-miR5368	0.000663775	0.002121027	5.798390508	up
gra-miR157a	3.41E-07	1.69E-06	3.251752874	up
gra-miR157b	3.41E-07	1.69E-06	3.251752874	up
hbr-miR156	3.41E-07	1.69E-06	3.251752874	up
hbr-miR159a	5.53E-43	3.08E-41	-1.683680957	down
hbr-miR6483	0.005596514	0.015558309	1.728819866	up
hbr-miR6485	1.85E-23	4.29E-22	6.811840804	up
lja-miR167	2.17E-09	1.89E-08	3.44841405	up
lus-miR156h	2.64E-06	1.10E-05	3.413721503	up
mdm-miR156b	0.014973863	0.035578923	5.108656101	up
mdm-miR156i	0.003694689	0.010480852	5.516131617	up
mdm-miR162a	8.05E-09	5.89E-08	-1.332251957	down
mdm-miR162b	8.05E-09	5.89E-08	-1.332251957	down
mdm-miR482a-5p	3.73E-08	2.53E-07	1.196719286	up
mes-miR156c	0.003694689	0.010480852	5.516131617	up
mes-miR156g	0.003694689	0.010480852	5.516131617	up
mes-miR156h	2.64E-06	1.10E-05	3.413721503	up
mes-miR156i	2.64E-06	1.10E-05	3.413721503	up
mes-miR156j	2.64E-06	1.10E-05	3.413721503	up
mes-miR162	1.10E-08	7.66E-08	-1.316035336	down
mtr-miR156b-5p	0.003694689	0.010480852	5.516131617	up
novel_miR_1	0.000146536	0.000522268	-1.192688276	down
novel_miR_101	0.000146536	0.000522268	-1.192688276	down
novel_miR_103	0.000540979	0.001748746	-4.971472581	down
novel_miR_104	4.69E-05	0.00017848	1.68936604	up
novel_miR_106	1.54E-10	1.47E-09	5.764200312	up
novel_miR_108	0.010729158	0.026871226	-1.519356369	down
novel_miR_11	1.23E-11	1.37E-10	-1.179707766	down
novel_miR_113	5.78E-10	5.18E-09	24.22109721	up
novel_miR_114	1.23E-11	1.37E-10	-1.179707766	down
novel_miR_119	1.42E-14	3.04E-13	4.263826404	up
novel_miR_12	0.014637409	0.035384346	-2.321336554	down

novel_miR_122	0.010290118	0.026871226	1.710684941	up
novel_miR_123	1.24E-40	4.30E-39	-1.996301057	down
novel_miR_124	0.001680009	0.005189362	-2.972584353	down
novel_miR_13	1.24E-40	4.30E-39	-1.996301057	down
novel_miR_134	3.83E-07	1.81E-06	6.68671792	up
novel_miR_135	1.23E-11	1.37E-10	-1.17993222	down
novel_miR_136	7.30E-14	1.35E-12	-2.923121839	down
novel_miR_138	5.82E-34	1.80E-32	4.889746316	up
novel_miR_140	3.33E-12	4.62E-11	4.051909401	up
novel_miR_145	2.28E-07	1.44E-06	2.709054585	up
novel_miR_149	0.000954415	0.003015083	-4.86536679	down
novel_miR_152	2.70E-06	1.10E-05	-6.134781208	down
novel_miR_153	0.011080417	0.027503177	-1.624503341	down
novel_miR_158	4.24E-06	1.71E-05	8.050499078	up
novel_miR_159	1.59E-13	2.76E-12	-2.058580573	down
novel_miR_16	3.79E-07	1.81E-06	2.716029151	up
novel_miR_161	2.63E-12	4.06E-11	-1.188904301	down
novel_miR_162	7.30E-11	7.25E-10	4.364117809	up
novel_miR_165	7.18E-45	4.99E-43	-2.786029357	down
novel_miR_166	1.15E-40	4.30E-39	-1.996766451	down
novel_miR_167	1.05E-49	9.69E-48	-1.616405892	down
novel_miR_168	0.000357337	0.001226416	-1.194649675	down
novel_miR_181	5.00E-10	4.64E-09	2.517433236	up
novel_miR_184	0.000163586	0.000575656	6.663578647	up
novel_miR_185	1.05E-49	9.69E-48	-1.616405892	down
novel_miR_198	0.010290118	0.026871226	1.710684941	up
novel_miR_200	0.010729158	0.026871226	-1.519356369	down
novel_miR_205	3.79E-07	1.81E-06	2.716029151	up
novel_miR_214	3.82E-08	2.53E-07	-1.468972566	down
novel_miR_216	7.30E-11	7.25E-10	4.364117809	up
novel_miR_217	0.000518805	0.001696799	-2.979720053	down
novel_miR_219	0.000146536	0.000522268	-1.192688276	down
novel_miR_220	4.48E-14	8.89E-13	-1.249801208	down
novel_miR_229	0.002067315	0.006315533	4.723226025	up
novel_miR_27	0.010290118	0.026871226	1.710684941	up
novel_miR_3	4.16E-12	5.51E-11	4.93858004	up
novel_miR_34	1.39E-11	1.49E-10	-1.176709191	down
novel_miR_42	1.23E-11	1.37E-10	-1.17993222	down
novel_miR_44	1.79E-31	4.53E-30	-1.971209243	down
novel_miR_50	1.05E-49	9.69E-48	-1.616405892	down
novel_miR_53	4.69E-05	0.00017848	1.68936604	up
novel_miR_54	1.79E-31	4.53E-30	-1.971209243	down
novel_miR_6	0.010729158	0.026871226	-1.519356369	down
novel_miR_63	0.000146536	0.000522268	-1.192688276	down

novel_miR_64	0.012593726	0.030982795	5.903458788	up
novel_miR_68	0.00038503	0.001305347	-2.917270653	down
novel_miR_73	0.010289302	0.026871226	3.637986682	up
novel_miR_76	0.001437122	0.004488989	-1.791469321	down
novel_miR_77	0.000146536	0.000522268	-1.192688276	down
novel_miR_78	9.20E-09	6.56E-08	-2.305171974	down
novel_miR_79	0.000515729	0.001696799	-1.47836932	down
novel_miR_8	0.000426647	0.001429011	-5.02021908	down
novel_miR_82	0.010729158	0.026871226	-1.519356369	down
novel_miR_83	0.010489995	0.026871226	-4.189828034	down
novel_miR_88	0.01389104	0.033874641	1.697608318	up
novel_miR_92	2.63E-12	4.06E-11	-1.188904301	down
novel_miR_98	3.33E-12	4.62E-11	4.051909401	up
ppe-miR156g	2.64E-06	1.10E-05	3.413721503	up
ptc-miR156g	3.41E-07	1.69E-06	3.251752874	up
ptc-miR156i	3.41E-07	1.69E-06	3.251752874	up
ptc-miR156j	3.41E-07	1.69E-06	3.251752874	up
rco-miR156c	0.003694689	0.010480852	5.516131617	up
rco-miR156g	3.41E-07	1.69E-06	3.251752874	up
rco-miR156h	3.41E-07	1.69E-06	3.251752874	up
rco-miR167c	0.008686346	0.023674552	1.892736812	up
stu-miR156g-5p	0.014973863	0.035578923	5.108656101	up
tcc-miR156b	0.003694689	0.010480852	5.516131617	up
tcc-miR156e	3.41E-07	1.69E-06	3.251752874	up
vun-miR162	8.05E-09	5.89E-08	-1.332251957	down
vvi-miR156g	3.41E-07	1.69E-06	3.251752874	up
vvi-miR156i	3.41E-07	1.69E-06	3.251752874	up
